# In-depth characterization of self-healing polymers based on π–π interactions

**DOI:** 10.3762/bjoc.17.166

**Published:** 2021-09-29

**Authors:** Josefine Meurer, Julian Hniopek, Johannes Ahner, Michael Schmitt, Jürgen Popp, Stefan Zechel, Kalina Peneva, Martin D Hager

**Affiliations:** 1 Laboratory of Organic and Macromolecular Chemistry (IOMC), Friedrich Schiller University Jena, Humboldstr. 10, 07743 Jena, Germany; 2 Jena Center for Soft Matter (JCSM), Friedrich Schiller University Jena, Philosophenweg 7, 07743 Jena, Germany; 3 Institute of Physical Chemistry (IPC), Friedrich Schiller University Jena, Helmholtzweg 4, 07743 Jena, Germany; 4 Abbe Center of Photonics (ACP), Friedrich Schiller University Jena, Albert-Einstein-Straße 6, 07745 Jena, Germany; 5 Leibniz Institute of Photonic Technology (IPHT), e. V. Jena, Albert-Einstein-Straße 9, 07745 Jena, Germany

**Keywords:** characterization of polymers, π–π-interactions, self-healing polymers, supramolecular polymers

## Abstract

The self-healing behavior of two supramolecular polymers based on π–π-interactions featuring different polymer backbones is presented. For this purpose, these polymers were synthesized utilizing a polycondensation of a perylene tetracarboxylic dianhydride with polyether-based diamines and the resulting materials were investigated using various analytical techniques. Thus, the molecular structure of the polymers could be correlated with the ability for self-healing. Moreover, the mechanical behavior was studied using rheology. The activation of the supramolecular interactions results in a breaking of these noncovalent bonds, which was investigated using IR spectroscopy, leading to a sufficient increase in mobility and, finally, a healing of the mechanical damage. This scratch-healing behavior was also quantified in detail using an indenter.

## Introduction

Damage inflicted on different materials is omnipresent. Consequently, nature established a mechanism dealing with this problem [1]. The regeneration after a damage is one of nature´s great abilities. For instance, a broken bone is healed [2] and sometimes even whole limbs can be regenerated as known from the amphib axolotl [3]. Additionally, nonliving natural materials can also be healed such as mussel byssus threads [4]. This specific process is based on reversible interactions, which are integrated in the chemical structure of the proteins of the thread [5]. Zinc–histidine metal complexes which are part of the protein’s structure enable the material to regenerate its mechanical performance after a damage event [1,6].

Besides the examples of self-healing/regeneration that exist in Nature, the general concept could also be transferred to different synthetic materials. Hereby, two concepts can be distinguished. In extrinsic self-healing materials, a material flow is achieved by the encapsulation of microcapsules [7] or microchannels [8] filled with liquid healing agent. In contrast, intrinsic self-healing [9] and, thus, regeneration of the materials without any additional required healing agents, can be obtained by the integration of dynamic covalent bonds or [10], as known from Nature, by supramolecular ones [11–12]. In previous studies, several of these interactions were already applied such as metal–ligand interactions [13–14], hydrogen bonds [15–16] or halogen bonds [17]. Furthermore, π–π interactions also feature a reversible behavior and were therefore utilized for the design of different self-healing polymers [18–20]. In this context, mainly the interaction between π-electron-deficient diimide groups and π-electron-rich pyrene moieties was applied resulting in a very strong and stable supramolecular bond [21–22]. The noncovalent interaction was found to be reversible and, therefore, enabled healing of scratches [18].

However, little is known about the exact healing mechanism on the molecular scale and the correlation to the macroscopic properties of such polymers. For this purpose, the current study will focus on the design of polymers containing π–π interactions and the quantification of the healing behavior as well as the in-depth characterization of the molecular behavior and the macroscopic properties, which reveals new insights into the self-healing materials based on π–π interactions.

## Results and Discussion

### Polymer synthesis

**Scheme 1:**
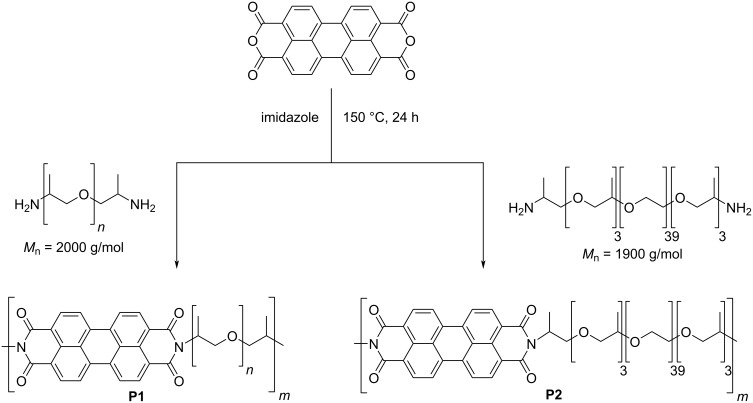
Schematic representation of the polymer synthesis of **P1** and **P2**.

For the synthesis of supramolecular polymers based on π-π interactions a literature reported procedure was utilized (see Scheme 1), which described the synthesis of polypropylene glycol-based polymers featuring aromatic diimides [23]. Following the procedure, perylene-3,4,9,10-tetracarboxylic dianhydride was converted with poly(propylene glycol) bis(2-aminopropyl ether) with a molar mass of approximately 2000 g/mol resulting in polymer **P1**. In order to study the influence of the polymer backbone on the material’s properties, the diamine containing polymer was exchanged to a triblock copolymer of poly(propylene glycol)-*block*-poly(ethylene glycol)-*block*-poly(propylene glycol) (PPG_3_-PEG_39_-PPG_3_) featuring also two amine groups as end groups. The molar mass of this reactant was 1900 g/mol. The conversion with perylene-3,4,9,10-tetracarboxylic dianhydride resulted in polymer **P2**. In both synthesis protocols imidazole was applied as a catalyst in order to obtain higher molar masses.

Subsequently, both polymers were characterized regarding their structure. For this purpose, size exclusion chromatography (SEC) was performed revealing a molar mass of *M*_n_ = 11,400 g/mol for **P1** and *M*_n_ = 17,400 g/mol for **P2** with respect to a PEG-standard. The SEC traces of both polymers are depicted in Supporting Information File 1. Furthermore, the polymers were analyzed using NMR spectroscopy. Herein, all signals could be assigned to both moieties within the polymers, the perylene and the polymer backbone. All spectra are shown in Supporting Information File 1.

### Characterization of the polymers

**Figure 1:**
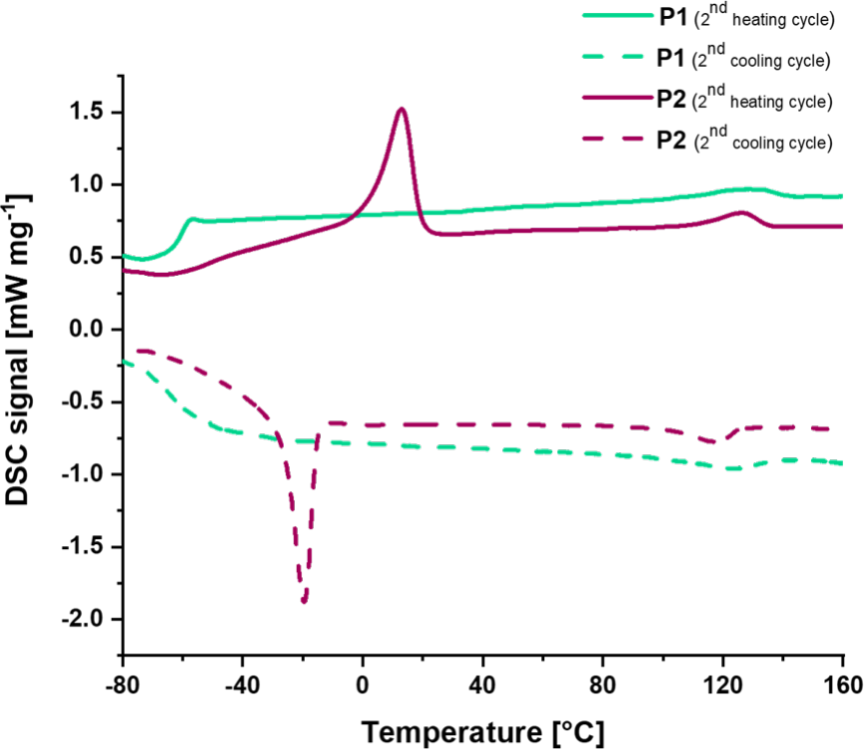
DSC-analysis of the polymers **P1** and **P2** (second heating and cooling cycle; 20 K/min for heating and cooling) with a glass transition temperature (*T_g_*) at −58 °C (**P1**) and −51 °C (**P2**) and the endothermic transition at 129 °C (**P1**) and 126 °C (**P2**). In addition, **P2** shows a *T_m_* at 13 °C.

After the synthesis of the polymers, the material and structural properties were analyzed in detail in order to study the molecular behavior and to correlate these results later with the healing behavior of the polymers. Firstly, the thermal properties of both polymers were investigated via differential scanning calorimetry (DSC) and thermogravimetric analysis (TGA). The TGA revealed a high thermal stability up to a temperature of 370 °C (for curves see Supporting Information File 1). The temperature was determined at a residual mass of 95%. The DSC on the other hand indicates several thermal transitions (see Figure 1). Both polymers feature a glass transition temperature (*T_g_*) at −58 °C (**P1**) and −51 °C (**P2**), respectively. Furthermore, the polymers have an endothermic transition at 129 °C (**P1**) and 126 °C (**P2**). This transition is associated with the activation of the perylene domains, which was also reported in literature [23]. During cooling, the reformation of the perylene domain was also observed. Finally, **P2** featured a second endothermic transition at 13 °C, which is based on the melting of the short PEG-block [24].

**Figure 2:**
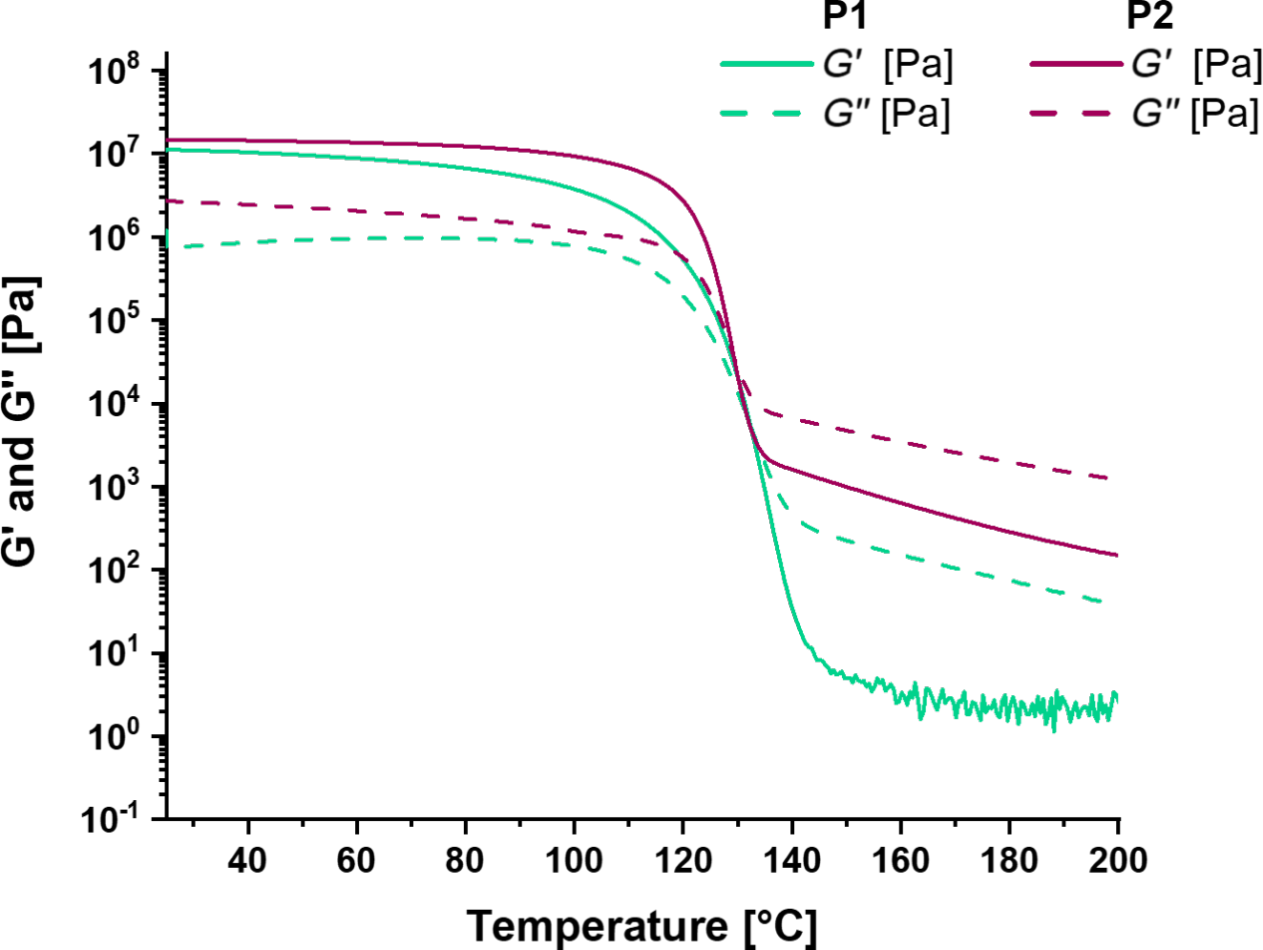
DMTA analysis of **P1** and **P2** showing the transition at around 130 °C due to the reversible π–π interactions.

Furthermore, both polymers were characterized via rheology and dynamic mechanical thermo-analysis (DMTA). The DMTA of **P1** and **P2** is depicted in Figure 2, revealing a network structure below temperatures of 120 °C. Above this temperature, a sharp transition (within a very small temperature range) and a significant drop of storage and loss modulus could be observed. Accordingly, this transition is associated with the endothermic signal measured in the DSC and based on the activation of the π–π interactions. Such a behavior could also be observed for other supramolecular polymers; however, the temperature window, in which the drop of the storage and loss moduli occurred, is rather small compared to other self-healing supramolecular polymers, e.g., metallopolymers [25].

**Figure 3:**
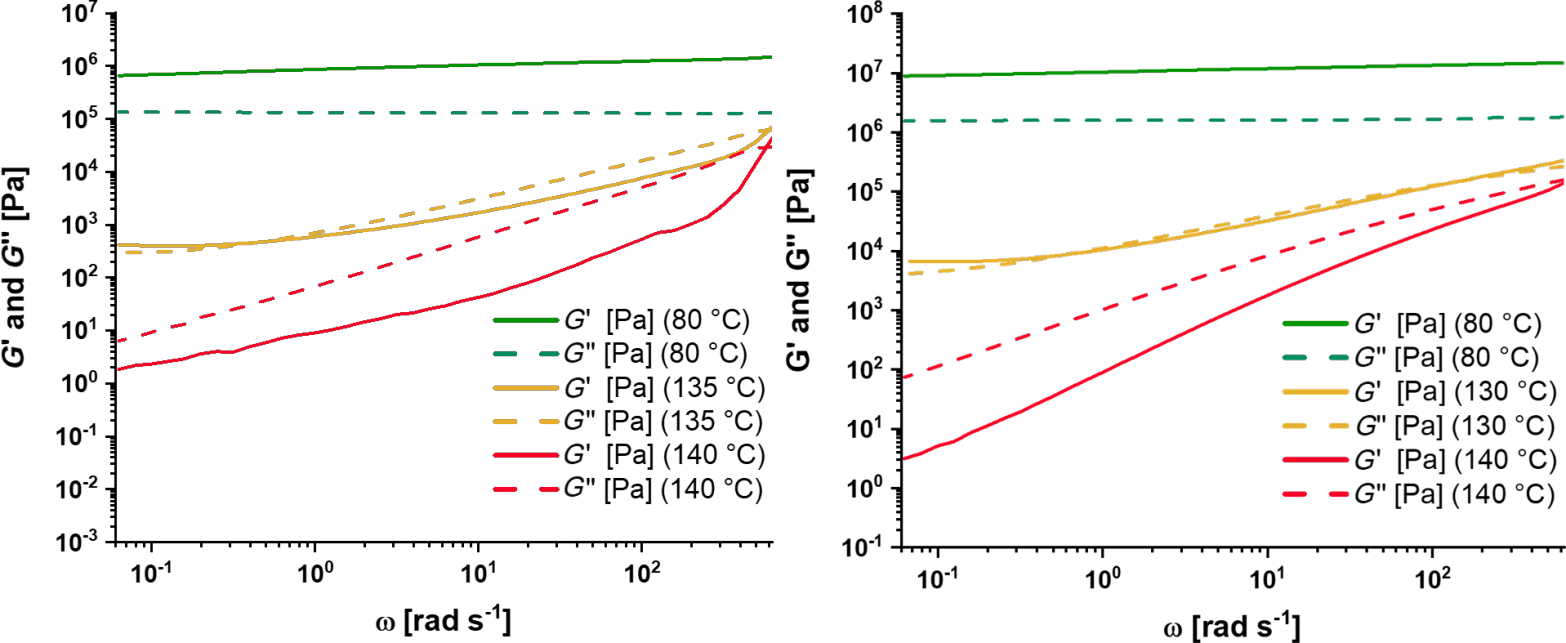
Frequency sweeps of polymers **P1** (left) and **P2** (right).

Supramolecular polymers feature certain temperature ranges, in which the noncovalent bond is activated. The degree of reversibility can be determined by the supramolecular bond lifetime [26]. For example, a study regarding ionomers revealed a strong correlation of the bond lifetime with the healing behavior [27]. A similar behavior was also observed for metallopolymers [13]. Consequently, the polymers **P1** and **P2** were also studied by frequency sweeps at certain temperatures (see Figure 3). At temperatures below the endothermic transition at 125–130 °C, no crossover of *G'* and *G''* could be observed indicating no active supramolecular bonds. Furthermore, at this temperature (80 °C) *G'* is higher than *G''* indicating a network structure of the polymers. This finding correlates with the DSC results, since the π–π interactions are not activated and, therefore, the polymer network is intact.

Within the transition, the frequency sweeps revealed a crossover of storage and loss modulus showing the activation of the π–π interactions, which goes hand in hand with the DSC results. Thus, a dynamic network structure could be revealed. The supramolecular bond lifetime was determined to be 15.87 s at 135 °C (**P1**) and 10.18 s at 130 °C (**P2**), respectively. The calculation was performed according to literature and Equation 1 [27].

[1] Supramolecular bond lifetime = 1/frequency (crossover)

However, at higher temperatures also no crossover could be observed, which is also in line with findings for other supramolecular bonds like ionic interactions [27]. In this temperature range, *G''* is higher than *G'* showing that the polymer is uncrosslinked. Thus, the mobility is very high, which is a precondition for the healing.

To get further insight into the molecular behavior of **P1** and **P2**, temperature dependent IR spectroscopy experiments of drop casted films of the respective polymers were carried out. The respective polymers were heated to 150 °C and an IR spectrum was recorded every 20 K. Afterwards the polymers were air cooled and further spectra at 100, 70, and 25 °C were recorded.

**Figure 4:**
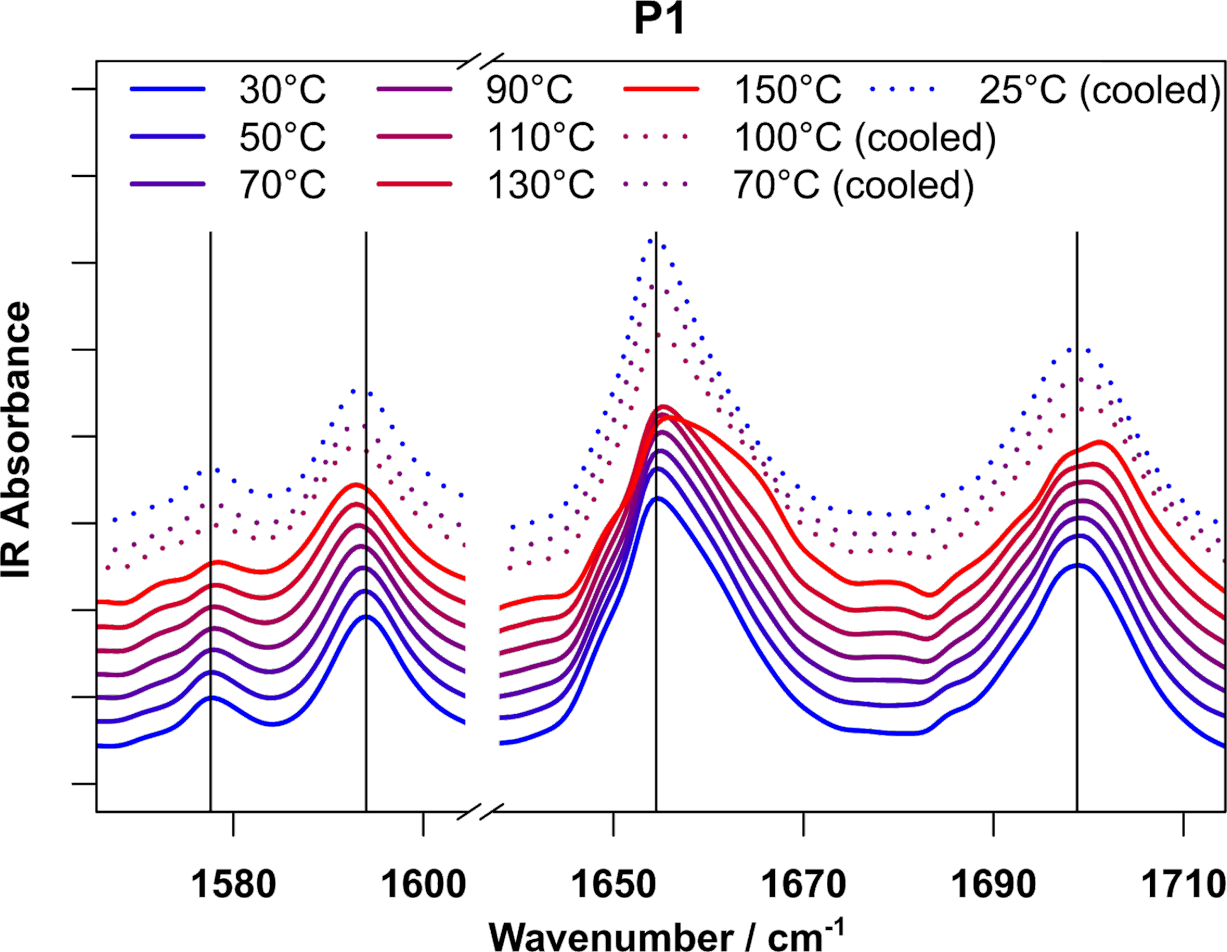
Temperature dependent IR spectra of **P1** drop casted on KBr in the C=C (1570–1605 cm^−1^) and C=O stretching region (1640–1710 cm^−1^). During heating, slight shifts in the position of all bands (1578 cm^−1^: +1 cm^−1^; 1594: −1.5 cm^−1^; 1656 cm^-1^: +0.8 cm^−1^; 1698: +1.5 cm^−1^. These shifts are partially reversed while cooling the polymer, indicating a reversible cause for the shifts. Additionally, all bands exhibit broadening during heating, especially noticeable at 150 °C, indicating a broader distribution of species contributing to the IR spectrum.

Figure 4 displays the aromatic C=C (1570–1605 cm^−1^) and C=O stretching (1640–1710 cm^−1^) region of the infrared spectra of **P1** recorded during heating. These regions are specific to the perylene moieties in the polymers and, therefore, allow a direct observation of the π–π interactions in the polymer. Both the C=C and C=O vibrations are sensitive to the electron density in the perylene systems, which changes depending on the strength of π–π interactions [28–30].

During heating, the C=C stretching vibrations located at 1578 and 1594 cm^−1^ show opposite behavior regarding their wavenumber position: While the band at 1578 cm^−1^ shifts to slightly higher wavenumbers (indicating more electron density in the perylene rings), the band at 1594 cm^−1^ shifts to slightly lower frequencies (indicating less electron density in the perylene rings). This seemingly counterintuitive behavior can be explained by the fact that the perylene-moieties act as both π-donors and -acceptors. Weakening of π–π interactions therefore results in higher electron densities in some part of the perylene moiety, while in other parts the electron density decreases. The C=O vibrations at 1656 and 1698 cm^−1^ on the other hand both shift to higher wavenumbers, indicating a strengthening of the carbon-oxygen bond. This is caused by a weakening of inter-perylene C–H–O interactions that also contribute to the stacking behavior [28–29], which in turn increases the electron density in the C=O bond.

In addition to these shifts in band positions, all bands show noticeable broadening during heating, which is most significant for the C=O vibration at 1656 cm^−1^. This indicates a broader distribution of species contributing to the IR spectrum, which is consistent with increased mobility of the perylene moieties which allows for more possible geometries. Furthermore, it is evident that the broadening of the band shows an intensive increase at 150 °C. This nonlinear behavior indicates a drastic change in molecular structure around this temperature range, which corresponds to the observed signals in the DSC measurements and the findings of the DMTA analysis. The slight difference in temperature can be explained by the different experimental setups (open system for IR measurements, closed system for DSC) and the different sample preparations as well as different applied heating rates.

For **P2,** the observations (see Supporting Information File 1) are generally the same; however; these changes are much weaker for lower temperatures. While for **P1** a slight band shift can be observed even for 50 and 70 °C, **P2** only shows noticeable shifts at higher temperature. This aspect is consistent with the higher rigidity of **P2** at lower temperatures, caused by the presence of a second phase transition of the PEG moieties observed in the DSC. Nevertheless, at 150 °C, **P2** also shows the clear broadening of bands, which is consistent with the very similar positions for the perylene π–π interaction signal in the DSC.

All these findings clearly support that at increased temperatures the perylene–perylene π–π interactions are significantly weakened, which increases the mobility of the chains. Furthermore, the reversible nature of these processes is indicated by the recovery of the band shifts and band broadenings upon cooling of the polymers to room temperature.

### Self-healing behavior

Finally, the healing behavior of the polymers was studied in detail. For this purpose, an established method was applied enabling the detailed analysis of the scratch healing behavior by investigating the volume of the scratch [31–32]. A scratch was introduced into the material by using an indenter, afterwards, the sample was twisted to 90° and the profile was measured using an indenter resulting in the possibility to calculate the volume of the scratch. The subsequent heating at a certain temperature (80 °C, 125 °C or 150 °C) resulted in a healing behavior, which was quantified afterwards by measuring the profile again.

**Figure 5:**
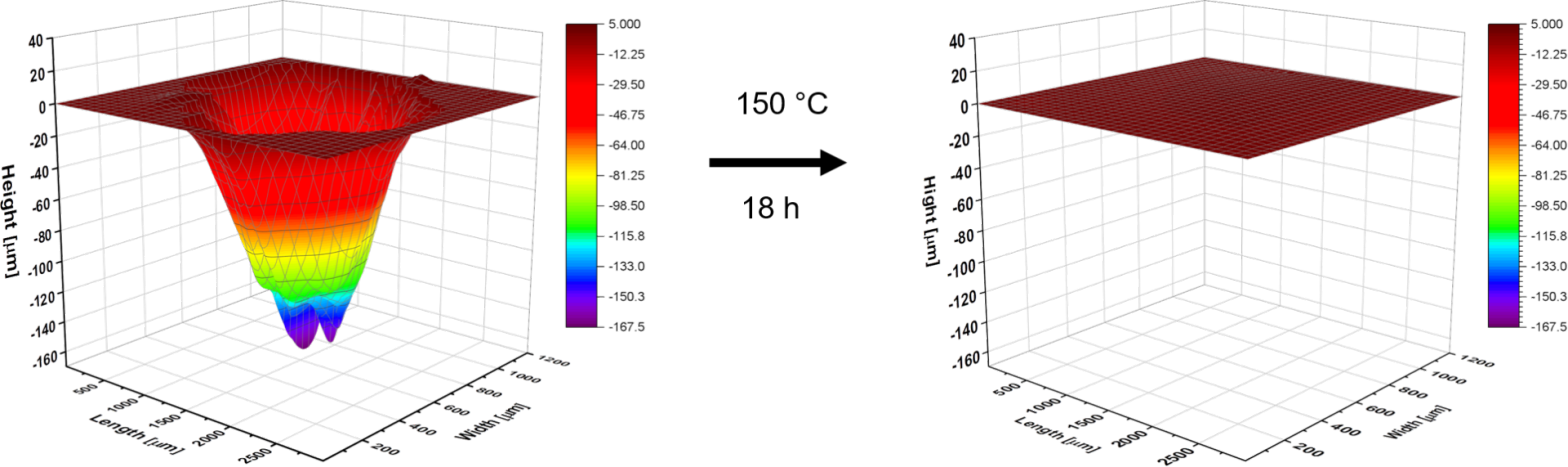
Schematic representation of the first healing of **P1** at 150 °C.

Using this approach, **P1** was studied first and the results are summarized in Table 1. For **P1**, a nearly complete healing at 150 °C was observed for the first scratch (see Figure 5) and a partial healing for the second scratch (see Supporting Information File 1). The healing behavior at 80 °C was significantly lower compared to 150 °C (see Supporting Information File 1) and the scratch could still be detected after 36 h at 80 °C. Furthermore, the healing was studied at 125 °C, which corresponds to the temperature, at which the change of the mechanical properties and flow behavior was started (see results of DMTA). Hereby, a partial healing was also observed (healing efficiency 50.64%, for pictures see Supporting Information File 1). Looking closer at the 3D-profiles, it can be seen that the depth of the scratch was reduced by more than 60% (from max. 64 µm to max. 23 µm). However, the width and the length of the scratch is nearly unchanged. Thus, the overall healing efficiency is lower compared to the reduction of the scratch depth.

**Table 1:** Overview of the healing of **P1**.

Scratch	Healing time/ temperature	Volume before healing [µm³]	Volume after healing [µm³]	Healing efficiency^a^

1	18 h; 150 °C	120,934,901	41,574	99.97%
2	18 h; 150 °C	33,233,081	8,757,225	73.65%
3^b^	18 h; 80 °C	23,741,395	12,086,157	49.09%
	18 h; 80 °C	12,086,157	11,194,985	7.37%
4	18 h; 125 °C	22,119,541	10,917,396	50.64%

^a^The healing efficiency was calculated based on a literature reported equation [31–32]. ^b^The third scratch was healed at 80 °C for 18 h and afterwards, it was further healed again for 18 h at 80 °C. The overall healing efficiency is 52.85%.

In contrast, the analysis of the healing behavior of **P2** was just impossible. The scratching of the material resulted in no measurable scratch, which is presumably associated with the melting of the PEG-block at temperatures below room temperature (see Supporting Information File 1). Thus, the material seems to feature a highly efficient elastic recovery (see Supporting Information File 1 for the “scratch” analysis) resulting in a (fast) crack closure behavior without the necessity of activating the supramolecular π–π interactions. Consequently, the healing of **P2** could not be analyzed in detail since the polymer backbone seems to influence the mobility behavior of the material significantly.

The observed healing behavior of **P1** goes hand in hand with the structural analysis of the material before. Since the healing behavior is based on π–π interactions, a sufficient healing could only be observed at temperatures above the activation of the π–π interactions. At temperatures below the activation, the healing was incomplete and presumably associated with the elastic recovery of the material. Consequently, for the first time, a correlation between the structure behavior of polymers featuring reversible π–π interactions and the healing behavior could be obtained.

## Conclusion

Supramolecular polymers based on π–π interactions were synthesized and characterized in detail. The mechanical and thermal behavior was studied revealing an activation of the supramolecular interactions at 125 °C. This finding could also be verified by temperature-depending IR-spectroscopy indicating a broadening of the aromatic signals at 150 °C, which correlates to the changes of the molecular structure. Furthermore, the scratch healing was analyzed in detail showing that only one of the two polymers studied, polymer **P1** is able to heal scratches in a sufficient manner at temperature higher than the activation of the π–π interaction. In contrast, polymer **P2** could not be damaged in a sufficient manner (under the utilized conditions) due to the polymer design. In particular, the poly(ethylene glycol) block resulted in a sufficient elastic recovery. Consequently, the material could not be analyzed via scratch testing in sufficient manner.

The current study reveals a strong correlation between the molecular structure of the supramolecular building units and the healing behavior of such polymers. Thus, the polymer backbone influences the healing behavior of the materials significantly and, consequently, this aspect is also highly important for the design of novel self-healing materials. However, further studies are required in order to understand the influence of the utilized polymer backbone in more detail.

## Experimental

### Materials and instrumentation

All chemicals were used as received from Sigma-Aldrich (Darmstadt, Germany) if not otherwise stated. The dialysis tubings were purchased from Spectrum Labs (Spectra/PorTM, pre-wetted tubing, 3.5 kDa) and were rinsed with water before use.

Nuclear magnetic resonance spectra were measured using a Bruker AC 300 (300 MHz) spectrometer at 298 K (Billerica, MA, USA). The chemical shift is given in parts per million (ppm on δ scale) related to a deuterated solvent.

Elemental analysis was performed utilizing a Vario El III (Elementar, Langenselbold, Germany).

Size exclusion chromatography measurements were performed utilizing the following setup: Shimadzu with CBM-20A (system controller), DGU-14A (degasser), LC-20AD (pump), SIL-20AHT (auto sampler), CTO-10AC vp (oven), SPD-20A (UV detector), RID-10A (RI detector), PSS SDV guard/1000 Å/1,000,000 Å (5 μm particle size) chloroform/isopropanol/triethylamine [94/2/4] with 1 mL/ min at 40 °C, poly(ethylene glycol) (standard).

Differential scanning calorimetry was measured on a Netzsch DSC 204 F1 Phoenix instrument (Selb, Germany) under a nitrogen atmosphere with a heating rate of 20 K min^−1^ (first and second heating cycle) and 10 K min^−1^ (third heating cycle). In general, the first cycle is used as annealing step, which deletes the thermal history of the sample, and thus is neglected.

Thermo gravimetric analysis was carried under nitrogen atmosphere using a Netzsch TG 209 F1 Iris (Selb, Germany) with a heating rate of 10 K min^−1^ from 25 to 600 °C. The thermo gravimetric analysis revealed degradation temperatures above 370 °C for all synthesized polymers.

All dynamic mechanical analysis (DMA) experiments were performed on a MCR 301 rheometer (SN80386674) from Anton Paar (Graz, Austria) using a convection temperature device CTD 450 which covers a broad temperature range of −100 to 450 °C. For measurements and the export of data the Rheocompas- software was used.

The temperature sweeps (DMTA) and frequency sweeps (FS) were measured with a plate-plate setup (D-PP15-SN0). The sample was heated to 150 °C and the sample gap was set to 1 mm. For the DMTA, the samples were cooled to 25 °C and heated up to 200 °C with a heating rate of 2 °C/min under a frequency (f) of 1 Hz with 1% shear strain (γ). For the frequency sweeps, the sample was firstly annealed at the desired temperature (80, 130 (**P2**) or 135 (**P1**) and 140 °C). Afterwards, the frequency was decreased in a logarithmic profile from 100 up to 0.01 Hz at a strain of 1%.

All infrared (IR) spectra were recorded on a Nicolet iS5 FTIR spectrometer (Thermo Fisher Scientific, Waltham, Massachusetts, United States of America), equipped with potassium bromide windows and beam splitter. The sample temperature was controlled with a temperature cell and temperature controller combination (TFC-M13-3 / ATC-024-1, Harrick Scientific Products, Pleasantville, New York, United States of America), which provides a heated sample chamber suitable for 12 mm windows.

To collect IR spectra of the samples, first KBr windows were prepared directly before the measurement by pressing 200 mg dry spectroscopic grade KBr (Merck KGaA, Darmstadt, Germany) into a ∅ 12 mm-form under vacuum and 7 MPa pressure. The window was subsequently transferred into the temperature cell and secured with a lockring and teflon seals. A background spectrum with 32 scans and 4 cm^−1^ spectral resolution was recorded. 1 mg of the respective sample was dissolved in 100 µL spectroscopic grade CHCl_3_ (Uvasol^®^, Merck KGaA, Darmstadt, Germany) and 20 µL drop casted onto the window. To record the IR spectra of the samples, the temperature cell was heated to the respective temperature (30–150 °C in 20 °C steps) and left to equilibrize for 2 minutes. Afterwards, a sample spectrum with 32 scans was recorded.

All graphics were generated with GNU R (version 4.0.2) [33] without further preprocessing of the spectra.

The preparation of the samples and the self-healing experiments (including evaluation of the data) were performed according to literature [31]. In the first step, the sample was hot pressed (at 150 °C, at about 2 t for 3 minutes) in a special manufactured mold. The pressed polymer samples were embedded in epoxy resin consisting of Epoxy Resin L and Hardener CL from R&G Faserverbundstoffe GmbH, followed by grinding of the sample with sandpaper (P60 to P2500).

The self-healing scratch tests were performed on an Anton Paar Micro scratch tester MST3 on a STeP 4 platform. The instrument was equipped with 10 µm and 50 µm Rockwell C indenters and the optical images were taken with the lenses MPlan N 5×/0.10/FN22. The scratches were performed with a 50 µm Rockwell indenter, 1500 mN normal force and 15 passages on a length of 2000 µm and a scratch speed of 30,000 µm/min. Subsequently, the scratch was imaged by the microscope in panorama mode. The sample was turned 90° in order to measure the profile of the scratch. Therefore, 150 scratches along the scratch (every 20 µm) were performed with a 10 µm Rockwell indenter with the following parameters: 3 mN normal force, 200 µm/min scratch speed, length 1600 µm (**P1**) or 600 µm (**P2**).

For the visualization and evaluation of the scratch profile data recorded by the MST3, a Python-based GUI controlled program was developed, which mainly uses the well-established data analysis library pandas as well as the SciPy and NumPy libraries for linear algebra, integration and interpolation.

### Polymer synthesis

The polymer synthesis was adapted from literature [23] and is reported briefly in the following.

**P1**: Perylene-3,4,9,10-tetracarboxylic dianhydride (1.02 g, 2.60 mmol), poly(propylene glycol) bis(2-aminopropyl ether) (*M*_n_ = 2000 g/mol; 5.2 g, 2.60 mmol) and imidazole (18 g, 264.39 mmol) were mixed in a round bottom flask under nitrogen atmosphere. Afterwards, the reaction mixture was heated to 150 °C for 17 h. After cooling to room temperature, water and chloroform were added to the mixture. The organic phase was washed two times with water, dried over sodium sulfate and concentrated. The residual was dissolved in tetrahydrofuran and dialyzed for three days with solvent exchange two times per day (MWCO: 3500 g/mol). After the solvent evaporation, a dark violet polymer could be obtained. ^1^H NMR (300 MHz, CDCl_3_) δ 8.74–8.45 (m, 8H, perylene-*H*), 4.27–3.12 (m, 143H, PPG: OC*H_2_*, C*H*), 1.46–0.81 (m, 140H, PPG: C*H_3_*) ppm; SEC (PEG standard): *M*_n_ = 11,400 g/mol, *M*_w_ = 27,600 g/mol, Ð = 2.43 (RI detector), *M*_n_ = 11,800 g/mol, *M*_w_ = 25,000 g/mol, Ð = 2.12 (UV detector); elemental analysis: found: C: 63.81, H: 9.06, N: 1.48; expected: C: 64.04, H: 9.04, N: 1.18.

**P2**: Perylene-3,4,9,10-tetracarboxylic dianhydride (1.02 g, 2.60 mmol), poly(propylene glycol)-*block*-poly(propylene glycol)-*block*-poly(propylene glycol) bis(2-aminopropyl ether) (*M*_n_ = 1900 g/mol; 4.94 g, 2.60 mmol) and imidazole (18 g, 264.39 mmol) were mixed in a round bottom flask under nitrogen atmosphere. Afterwards, the reaction mixture was heated to 150 °C for 17 h. After cooling to room temperature, water and chloroform were added to the mixture. The organic phase was washed two times with water and one time with brine, dried over sodium sulfate and concentrated. The residual was dissolved in a 1:1 mixture of chloroform and tetrahydrofuran and dialyzed for two days with solvent exchange two times per day (MWCO: 3500 g/mol). After the solvent evaporation, a dark violet polymer could be obtained. ^1^H NMR (300 MHz, CDCl_3_) δ 8.86–8.31 (m, 8H, perylene-*H*), 4.29–3.15 (m, 185H, PPG-*H* (OC*H_2_*, C*H*) PEG-*H* (OC*H_2_*)), 1.46–0.91 (m, 9H, PPG: C*H_3_*) ppm; SEC (PEG standard): *M*_n_ = 17,400 g/mol, *M*_w_ = 34,100 g/mol, Ð = 1.95 (RI detector), *M*_n_ = 18,200 g/mol, *M*_w_ = 35,100 g/mol, Ð = 1.92 (UV detector); Elemental analysis: found: C: 57.94, H: 7.97, N: 1.50; expected: C: 58.71, H: 8.21, N: 1.14.

## Supporting Information

File 1 Additional data.
