# Supplementary material for: In-depth characterization of self-healing polymers based on π–π interactions
Source: Beilstein J Org Chem. 2021 Sep 29;17:2496–504. doi: 10.3762/bjoc.17.166 (PMC8491711; doi:10.3762/bjoc.17.166)
Supplement: File 1 — Additional data. [file Beilstein_J_Org_Chem-17-2496-s001.pdf]

BEILSTEIN JOURNAL OF ORGANIC CHEMISTRY

Supporting Information

for

In-depth characterization of self-healing polymers based on π–π interactions

Josefine Meurer, Julian Hniopek, Johannes Ahner, Michael Schmitt, Jürgen Popp, Stefan Zechel, Kalina Peneva and Martin D. Hager

Beilstein J. Org. Chem. doi:

Additional data

License and Terms: This is a supporting information file under the terms of the Creative Commons Attribution License (https://creativecommons.org/licenses/by/4.0). Please note that the reuse, redistribution and reproduction in particular requires that the author(s) and source are credited and that individual graphics may be subject to special legal provisions.
The license is subject to the *Beilstein Journal of Organic Chemistry* terms and conditions: (https://www.beilstein-journals.org/bjoc/terms)

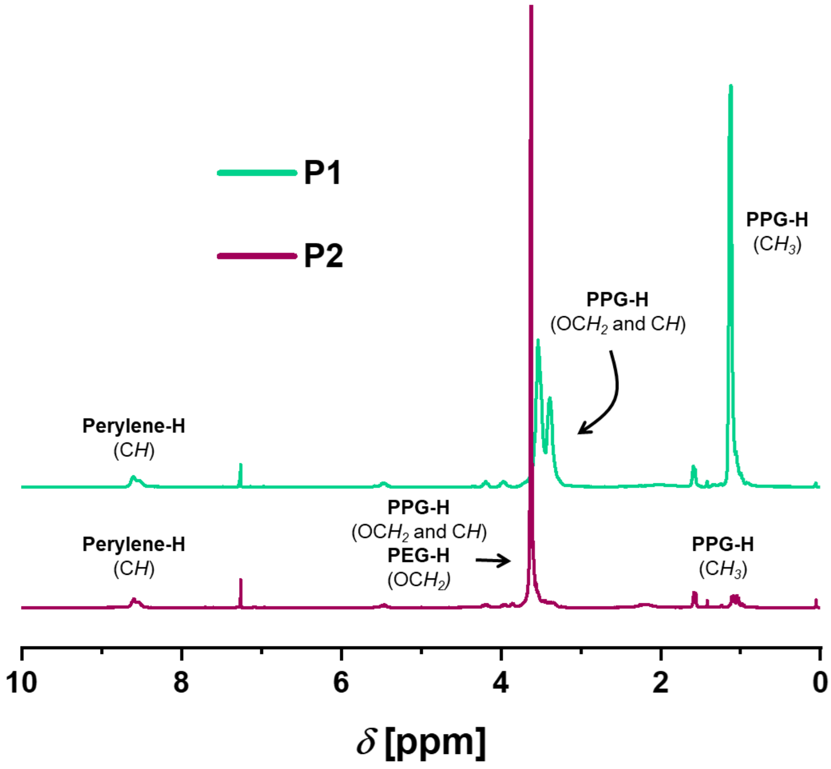

Figure S1: ^{1}H NMR spectra of **P1** and **P2** (300 MHz, $CDCl_3$).

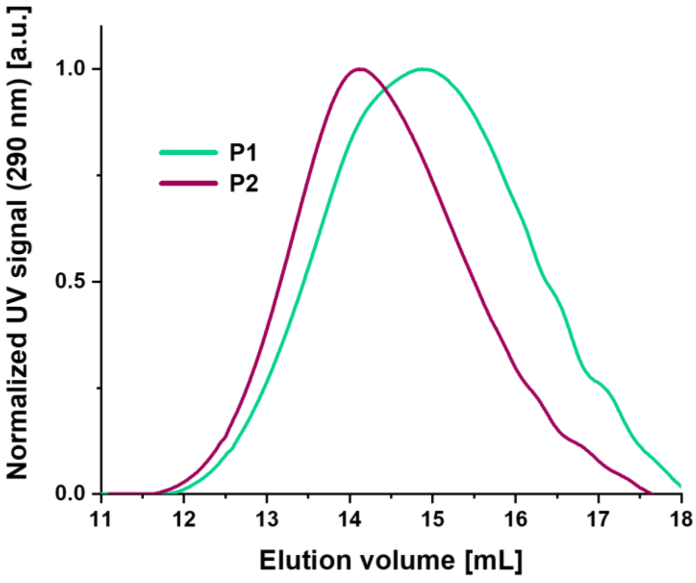

Figure S2: SEC-traces of the polymers **P1** and **P2** (chloroform/isopropanol/triethylamine [94/2/4]) revealing a molar mass of M_n = 11,400 g/mol for **P1** and M_n = 17,400 g/mol for **P2**.

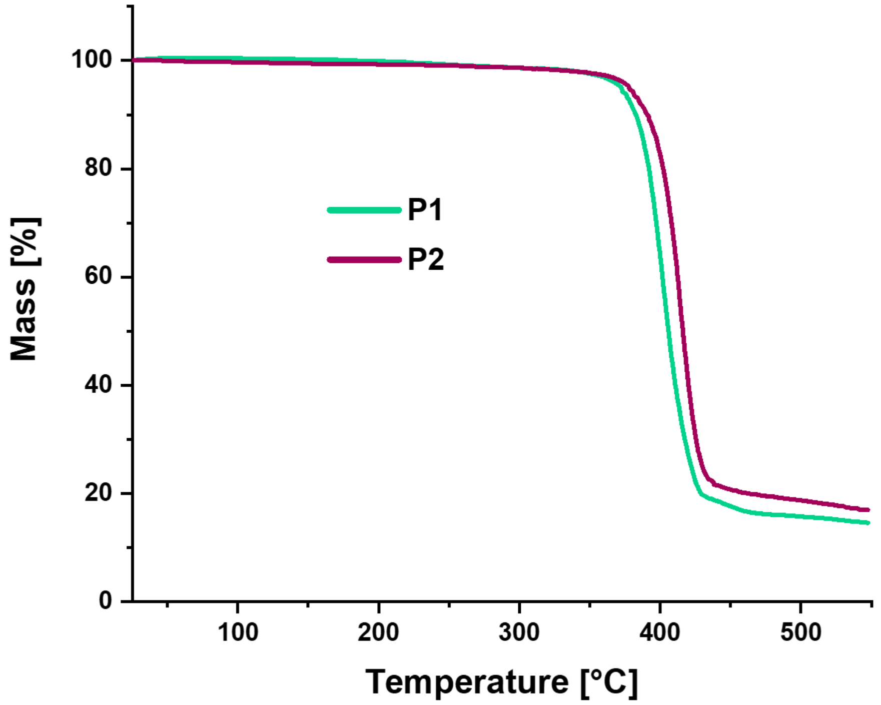

Figure S3: TGA analysis of **P1** and **P2** (heating rate 20 Kmin^{-1}).

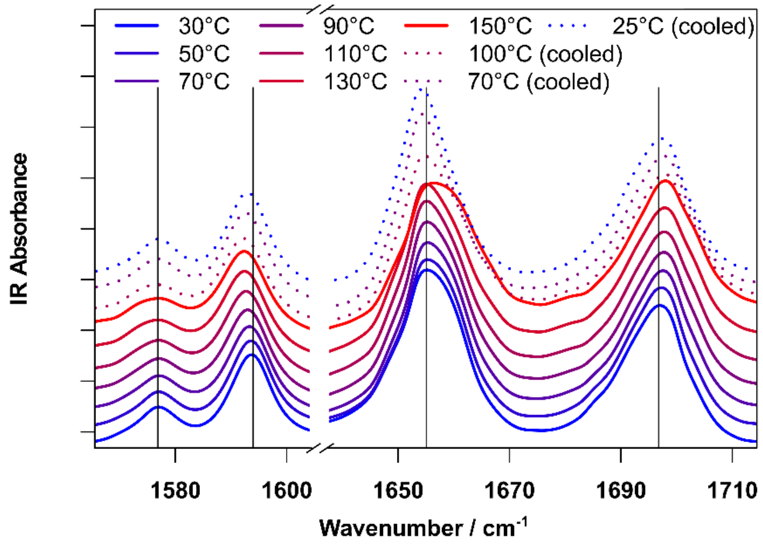

Figure S4: Temperature dependent IR spectra of **P2** drop casted on KBr in the C=C (1570–1605 cm^{-1}) and C=O stretching region (1640–1710 cm^{-1}). During heating, slight shifts in the position of all bands (1578 cm^{-1}: +0.2 cm^{-1}; 1595: −1.5 cm^{-1}; 1655 cm^{-1}: +0.4 cm^{-1}; 1696: +1.6 cm^{-1}). These shifts are partially reversed while cooling the

polymer, indicating a reversible cause for the shifts. Additionally, all bands exhibit broadening during heating, especially noticeable at 150 °C, indicating a broader distribution of species contributing to the IR spectrum

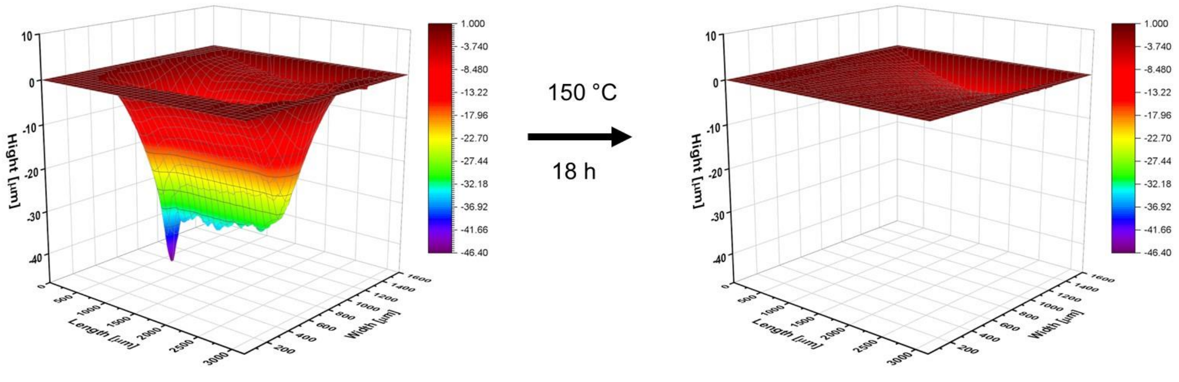

Figure S5: Self-healing of scratch two of **P1** at 150 °C for 18 h.

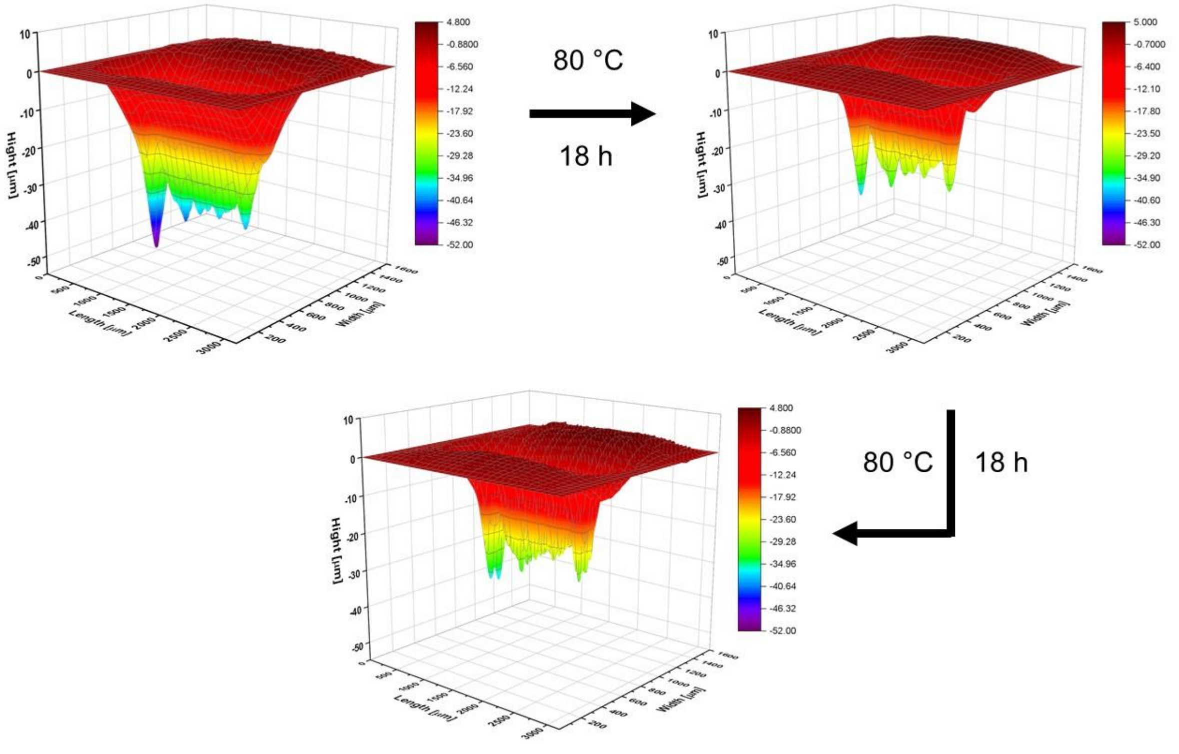

Figure S6: Self-healing of scratch three of **P1** at 80 °C for 18 h and additional 18 h.

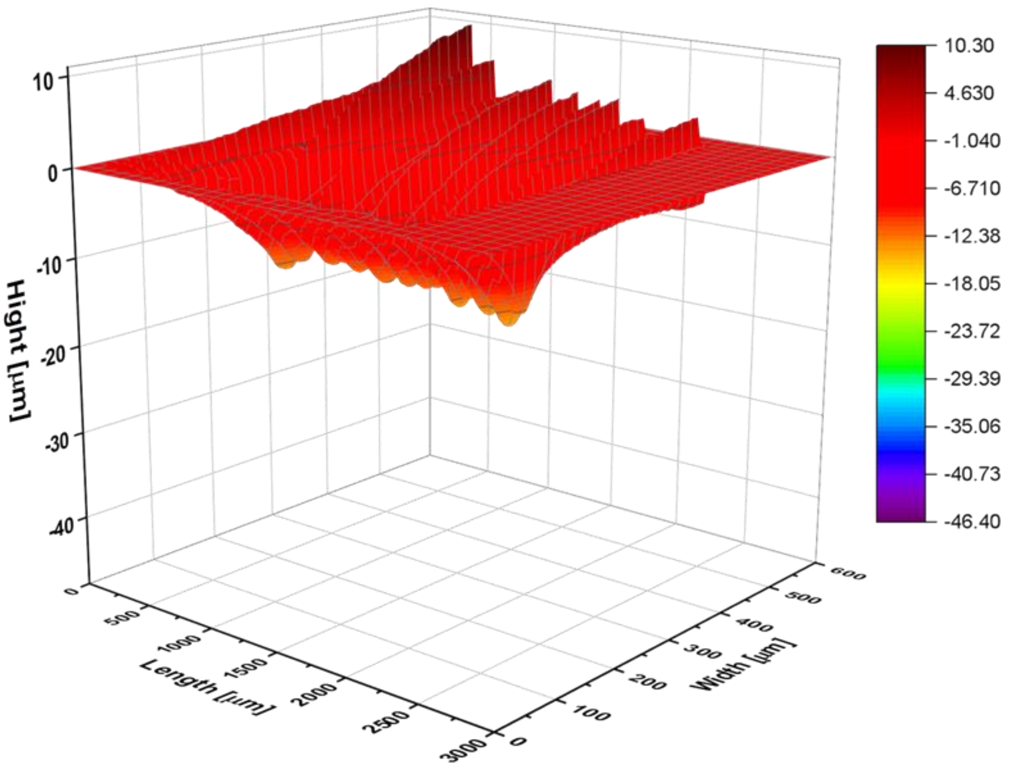

Figure S7: Analysis of the scratch of **P2** after indentation.

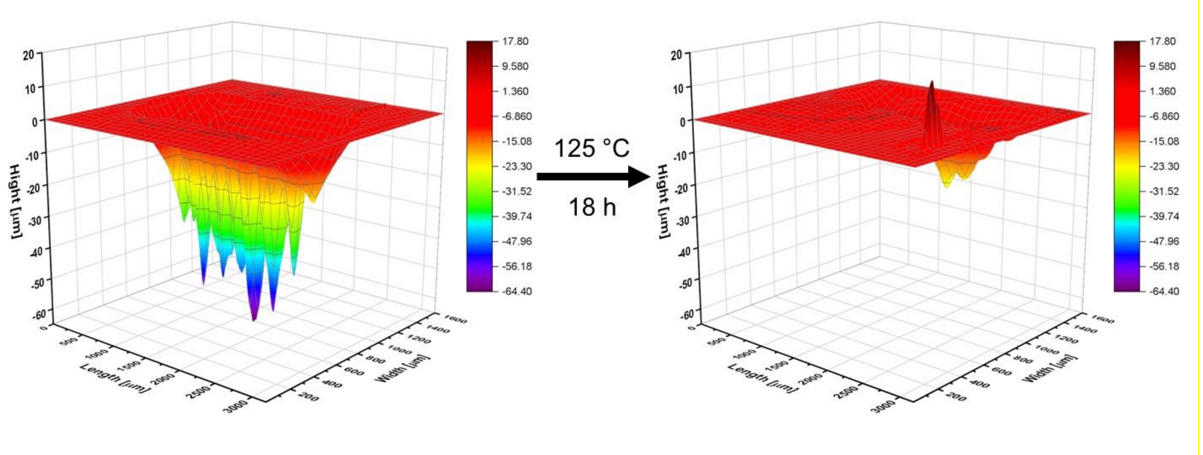

Figure S8: Self-healing of scratch three of **P1** at 125 °C for 18 h.
